#  Targeted point mutations of the m^6^A modification in miR675 using RNA-guided base editing induce cell apoptosis

**DOI:** 10.1042/BSR20192933

**Published:** 2020-05-05

**Authors:** Jindong Hao, Chengshun Li, Chao Lin, Yang Hao, Xianfeng Yu, Yidan Xia, Fei Gao, Ziping Jiang, Dongxu Wang

**Affiliations:** 1 Laboratory Animal Center, College of Animal Science, Jilin University, Changchun, China; 2 Department of Animal Science, Jilin Business and Technology College, Changchun, China; 3 Department of Hand Surgery, The First Hospital of Jilin University, Changchun, China

**Keywords:** cell apoptosis, gene expression, H19, m6A modification, miR675

## Abstract

Methylation of the adenine base at the nitrogen 6 position (m^6^A) is the most common post-transcriptional epigenetic modification of RNA, and it plays a very important role in regulating gene expression. To investigate the role of m^6^A methylation in the expression of non-coding RNA and miRNA, we used a system of adenine base editors (ABEs). Here, we mutated regions up- and downstream of miRNA 675 m^6^A modification sites in the *H19* locus using HEK293T, L02, MHCC97L, MHCC97H, A549, and SGC-7901 cells. Our results showed that a T–A base transversion had occurred in all cell lines. Moreover, mutation of the regions upstream of the miRNA 675 m^6^A modification site led to reduced expression of *H19* and the induction of cell apoptosis in HEK293T cells. To further confirm our results, L02 and MHCC97L cells were detected using ABEs system. The results indicated increased cell apoptosis and reduced expression of miR675 as well as *H19*. To confirm the relationship between *H19* and miR675 expression, overexpression and knockdown studies were performed. The results showed that reduced *HI9* expression induced cell apoptosis through miR675. Taken together, these results indicate that m^6^A modification can regulate the expression of *H19* and miR675 which induce cell apoptosis.

## Introduction

The long noncoding RNA (lncRNA) *H19* plays a crucial role in the development of cancer [1]. miR675, derived from exon 1 of *H19*, has been shown to have an oncogenic role in liver cancers [2,3]. Our previous data suggest that reduced expression of *H19* could induce cell apoptosis in A549, a lung cancer cell line [4]. Moreover, previous studies have shown that the *H19*/miR675 axis can regulate cell apoptosis [5]. These results demonstrate that altered expression of *H19* or miR675 can influence tumor cell behavior.

Recent reports have suggested that m^6^A (methylation of the adenine base at the nitrogen 6 position) methylation plays an important role in the post-transcriptional modification of RNA [6], and it is known that this modification is regulated by adenosine methyltransferases and demethylases [7,8]. As ‘writers,’ the m^6^A methyltransferases *METTL3, METTL14*, and *WTAP* methylate the N6 position of adenosine [9,10]. As ‘erasers,’ the m^6^A demethylases *FTO* and *ALKBH5* reverse the RNA methylation process [11,12]. Finally, *YTHDF2*, as an m^6^A ‘reader,’ recognizes m^6^A sites on target mRNAs and regulates the mRNAs’ fate [13–15]. Indeed, there is evidence that m^6^A modification in microRNA and lncNA affects cell development and fate [16]. These data indicate that m^6^A modification might have a role in noncoding RNA as well as miRNA.

Currently, the CRISPR/Cas9 system is the most widely used gene-editing tool. It uses an RNA-guide Cas9 protein combined with a short RNA (sgRNA) to induce double-strand breaks in target genomic DNA [17]. The adenine base editors (ABEs) system, which is based on the CRISPR/Cas9 platform, efficiently converts targeted A•T base pairs into G•C [18]. In the present study, the ABE7.10 system was used to analyze m^6^A modification of miRNA 675 in the *H19* locus. Moreover, cell apoptosis and m^6^A expression levels were evaluated in HEK293T, L02, and MHCC97L cells. The role of m^6^A modification in the expression patterns of miRNA and lncRNA was analyzed using the ABEs system.

## Materials and methods

### Cell culture

HEK293T, L02, MHCC97L, MHCC97H, SGC-7901, and A549 cells were cultured in Dulbecco’s modified Eagle’s medium, high glucose (Gibco, U.S.A.), supplemented with 10% fetal bovine serum (Gibco, U.S.A.). The cells were maintained at 37°C in 5% CO_2_.

### Construction and transfection of the plasmids

ABE7.10 plasmids were obtained from Addgene (102919). The m^6^A modification of miR675 in the *H19* locus (upstream of position: chr11:2018320 and downstream of position: chr11:2017630) was analyzed using the online software tool m6AVar (http://m6avar.renlab.org).

Protocols for sgRNA design and the procedures required for *in vitro* transcription have been described previously [17]. The sgRNA-oligo sequences used in the present study are listed in Supplementary Table S1.

For cell transfection, HEK293T, L02, MHCC97L, MHCC97H, SGC-7901, and A549 cells were seeded into 48-well poly-d-lysine-coated plates (Corning) in the absence of any antibiotic. Twelve to fifteen hours after plating, cells were transfected with 750 ng of base-editor plasmid and 250 ng of guide RNA plasmid in the presence of 1 µl of Lipofectamine 2000 (Thermo Fisher Scientific).

### Knockdown and overexpression of *H19* and miR675

Synthetic RNA oligonucleotides targeting *H19* were obtained from RiboBio (Guangzhou, China). The siRNA target sequence was GCGGGTCTGTTTCTTTACT. pcDNA3.1-H19 was purchased from GenePharma (Shanghai, China). miR675-3p-mimics and miR675-3p-inhibitor were obtained from RiboBio (Guangzhou, China). HEK293T cells were transfected with si-H19, pcDNA3.1-H19, miR675-3p-mimics, and miR675-3p-inhibitor for 48 h, respectively. Control cells were transfected with nonspecific, scrambled siRNA.

### Gene expression analysis

Total RNA was extracted from cells using the AllPrep DNA/RNA Micro Kit (QIAGEN, Germany) according to the manufacturer’s instructions. cDNA was synthesized using the First-Strand cDNA Synthesis kit (Promega, U.S.A.). Quantitative real-time PCR (qRT-PCR) was performed to determine *H19*, miR675, and m^6^A-related gene expression using the BioEasy SYBR Green I Real-Time PCR Kit on Bio-Rad iQ5 Multicolor Real-Time PCR Detection System (Bioer Technology, China). The miR675 3p and 5p sequences are listed in Supplementary Table S2, and the miRNA primer sequences are listed in Supplementary Table S3. The primer sequences of m^6^A-related genes and *H19* are listed in Supplementary Table S4. For PCR, the initial denaturation was conducted at 95°C for 3 min, followed by 40 cycles of denaturation at 95°C for 10 s, annealing at 60°C for 15 s, and extension at 72°C for 30 s. The 2^−ΔΔ*C*_T_^ method was used to determine relative gene expression. The experiments were performed at least in triplicates.

### Cell apoptosis analysis

The procedure for cell apoptosis detection has been previously described [19]. Briefly, HEK293T, L02, and MHCC97L cells were used for Annexin V-FITC/PI staining after treatment with ABE7.10 plasmids, si-H19, pcDNA3.1-H19 and miR675-3p-mimics and inhibitor for 48 h. Following incubation, the cells were washed twice with PBS and pooled at a concentration of 1 × 10^6^ cells/ml. For each treated cell sample, Annexin V-FITC and PI were added according to the manufacturer’s instructions. These cells were incubated for 30 min and then analyzed with an Accuri™ C6 flow cytometer (BD Biosciences, Franklin Lakes, NJ, U.S.A.).

### Immunofluorescence staining

Briefly, the cells were washed three times in PBS and then fixed with 4% paraformaldehyde for 30 min at room temperature. After fixation, the cells were washed again with PBS containing 0.2% Triton X-100 for 30 min. The cells were then incubated in PBS containing 1% bovine serum albumin (BSA) for 1 h. Next, the cells were probed with m^6^A (1:500, Abcam) antibodies and incubated at 4°C overnight. Following this, the cells were washed three times with PBS for 10 min each followed by incubation with Alexa Fluor 488–conjugated secondary (anti-rabbit) antibodies for 1 h at room temperature. DNA was stained with 10 ng/ml Hoechst 33342 (Thermo Scientific) for 15–20 min. The cells were then washed thrice with PBS for 10 min each, air-dried, and mounted on a coverslip and a glass slide using an antifade mounting medium (BOSTER, China). A confocal laser scanning microscope was used for imaging.

### Statistical analysis

All data were analyzed using GraphPad Prism 5.0 (GraphPad Software, Inc., San Diego, CA). A *t* test (Unpaired *t* test) was used to analyze the data. A *P-*value <0.05 was considered statistically significant.

## Results

### Targeted point mutations of m^6^A modification sites induce cell apoptosis

**Figure 1:**
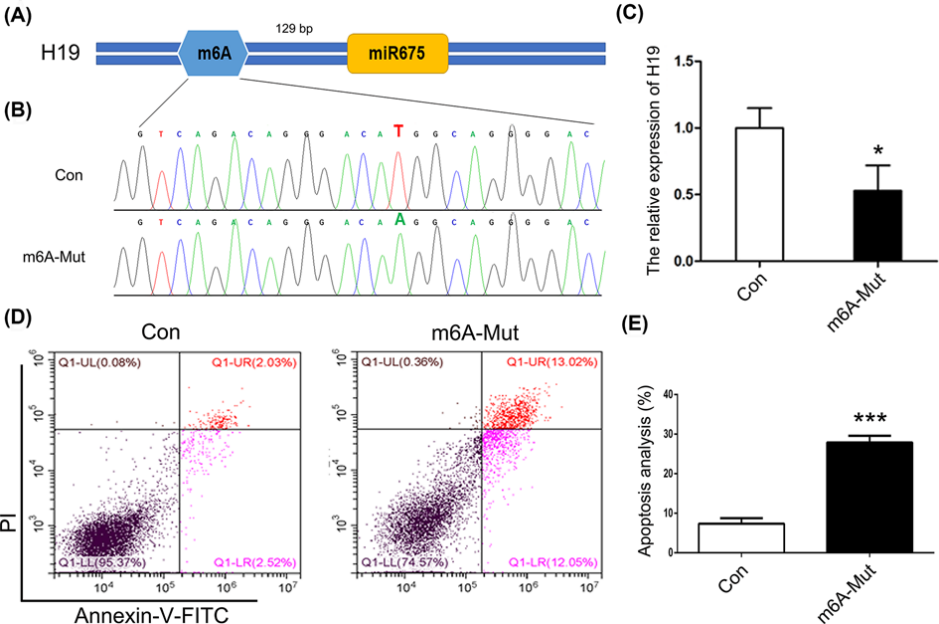
The role of m^6^A modification upstream of miR675 (**A**) The schematic of m^6^A modification. (**B**) Sequencing analysis of the m^6^A modification site. (**C**) *H19* expression analyzed by qPCR. (**D**) Cell apoptosis was analyzed after mutation of the m^6^A modification site. (**E**) Statistical analysis of apoptotic cell percentage. The data are presented as the mean ± SD. * (*P*<0.05) and *** (*P*<0.005) indicate statistically significant differences.

**Figure 2:**
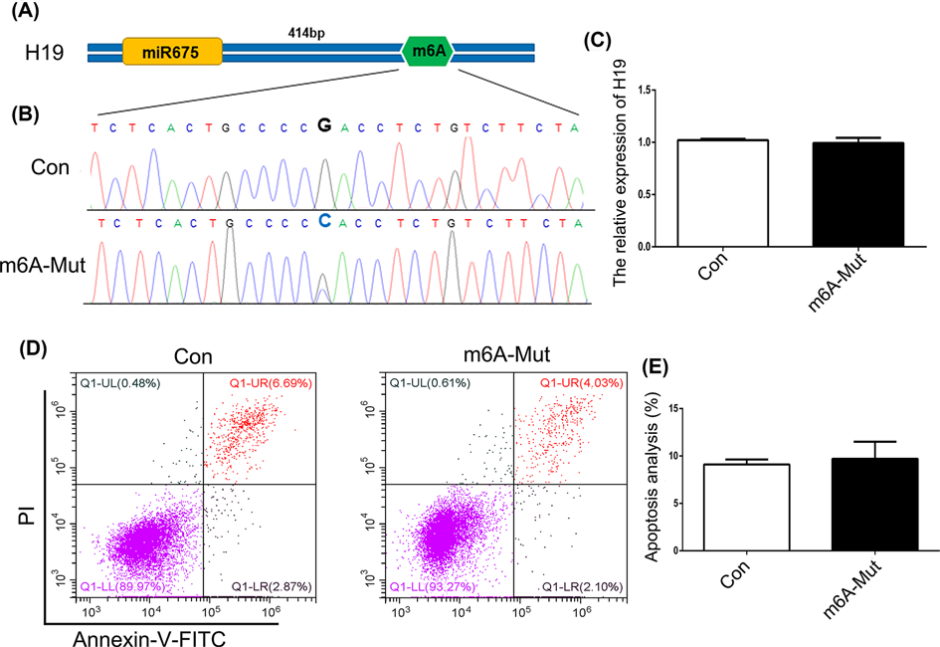
The role of m^6^A modification downstream of miR675 (**A**) The schematic of m^6^A modification. (**B**) Sequencing analysis of the m^6^A modification site. (**C**) *H19* expression was analyzed by qPCR. (**D**) Cell apoptosis was analyzed after mutation of the m^6^A modification site. (**E**) Statistical analysis of apoptotic cell percentage. The data are presented as the mean ± SD.

**Figure 3:**
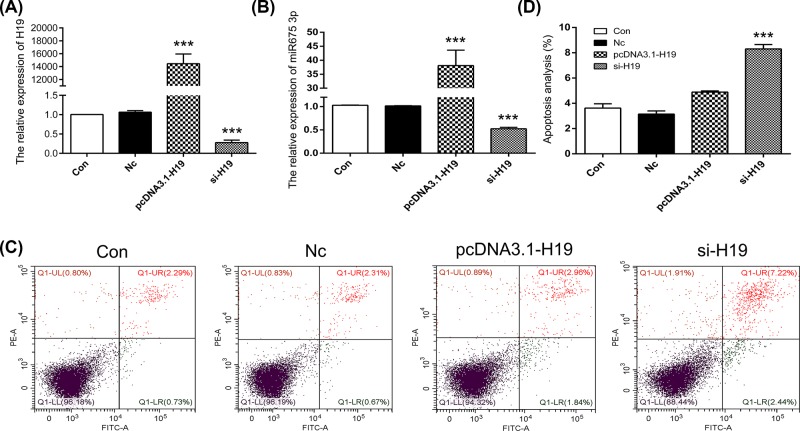
The expression pattern of *H19* in apoptosis Expression of *H19* (**A**) and miR675-3p (**B**) were analyzed by qPCR. (**C**) Cell apoptosis was analyzed after mutation of the m^6^A modification site. (**D**) Statistical analysis of apoptotic cell percentage. The data are presented as the mean ± SD. *** (*P*<0.005) indicates a statistically significant difference.

To investigate the role of m^6^A modification in the expression of *H19*, the ABE7.10 system was used. The m^6^A modification site 129 bp upstream of miR675 in the *H19* locus was mutated in HEK293T cells (Figure 1A). Results of Sanger sequencing suggested T–A base transversion (Figure 1B). To confirm these results, similar tests were carried out with MHCC97H, SGC-7901, and A549 cells. The results confirmed T–A base transversion (Supplementary Figure S1). qPCR results showed decreased expression of *H19* in the m^6^A-Mut group compared with that in the Con group (Figure 1C). To decipher the biological impact of m^6^A modification, we examined cell apoptosis. Our results indicated an increased apoptosis rate in the m^6^A-Mut group (Figure 1D,E). To further confirm the importance of m^6^A modification to *H19* as well as to miR675, we mutated the m^6^A modification site 414 bp downstream of miR675 (Figure 2A). Results of Sanger sequencing suggested G–C base transversion (Figure 2B). qPCR results showed no difference in *H19* expression between the Con and m^6^A-mut groups (Figure 2C). Moreover, the cell apoptosis rate did not increase after point mutations of the m^6^A modification sites (Figure 2D,E). To further analyze *H19* expression patterns in apoptotic cells, knockdown or overexpression of *H19* was performed in HEK293T cells. The results showed that *H19* expression was reduced after transfection with si-H19 and was increased following transfection with pcDNA3.1-H19 (Figure 3A). miR675-3p expression was analyzed by qPCR. The results showed that the miR675-3p expression was similar to the expression pattern of *H19* (Figure 3B). The cell apoptosis results showed that reduced expression of *H19* induced cell death (Figure 3C,D). These results indicate that m^6^A modification regulates *H19* expression which may induce cell apoptosis.

### Targeted point mutations of m^6^A modification sites in liver cancer cells

**Figure 4:**
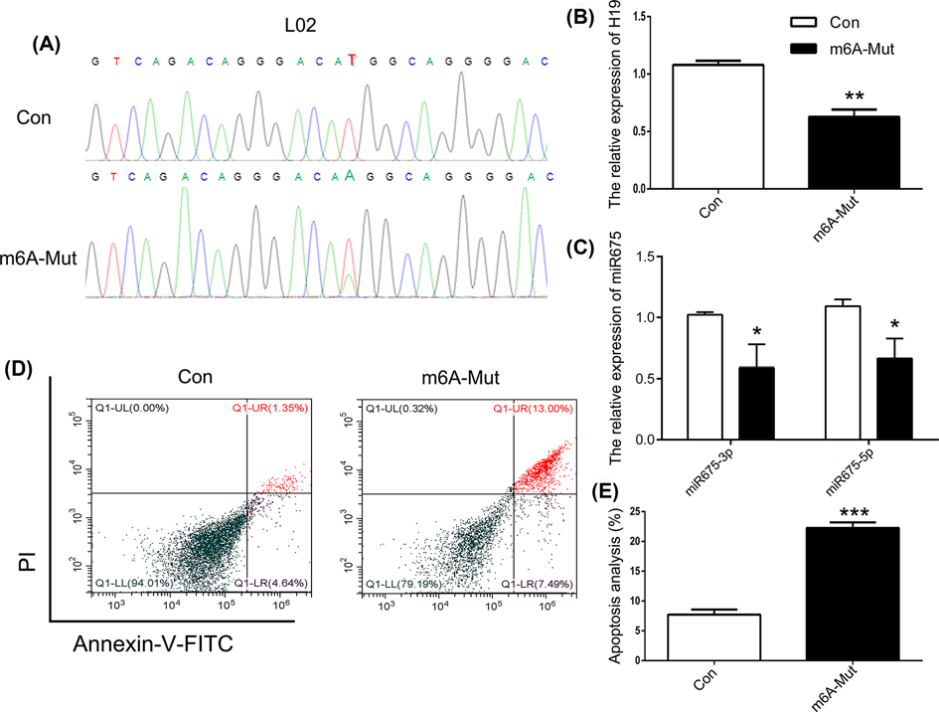
Mutated m^6^A modification of miR675 expression in L02 cells (**A**) Sequencing analysis of m^6^A modification site. The expression of *H19* (**B**) and miR675 (**C**) were analyzed by qPCR. (**D**) Cell apoptosis was analyzed after mutation of the m^6^A modification site. (**E**) Statistical analysis of apoptotic cell percentage. The data are presented as the mean ± SD. * (*P*<0.05), ** (*P*<0.01) and *** (*P*<0.005) indicate statistically significant differences.

**Figure 5:**
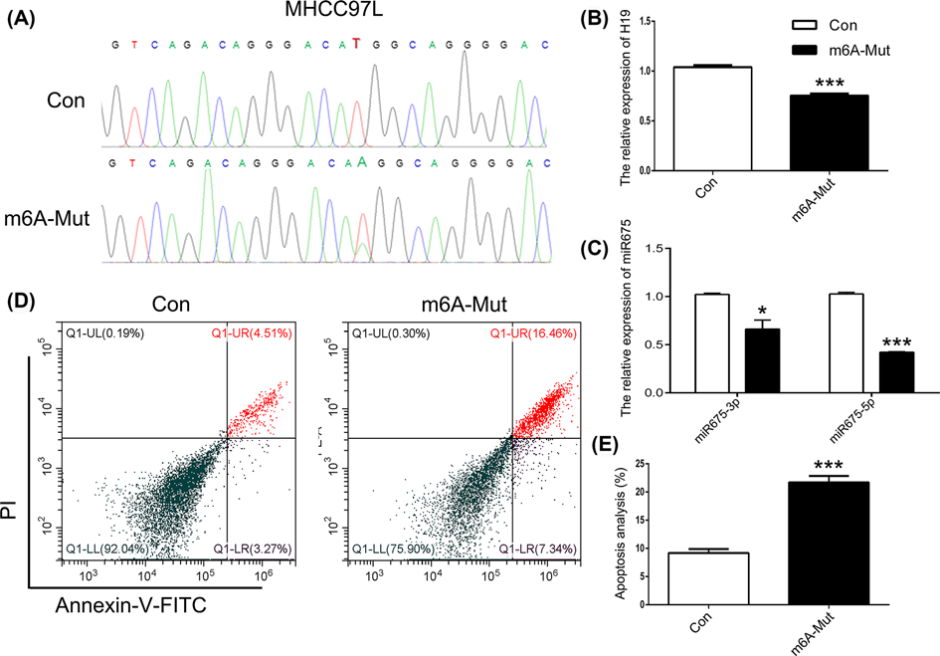
Mutated m^6^A modification of miR675 in MHCC97L cells (**A**) Sequencing analysis of the m^6^A modification site. The expression of *H19* (**B**) and miR675 (**C**) were analyzed by qPCR. (**D**) Cell apoptosis was analyzed after mutation of the m^6^A modification site. (**E**) Statistical analysis of apoptotic cell percentage. The data are presented as the mean ± SD. * (*P*<0.05) and *** (*P*<0.005) indicate statistically significant differences.

**Figure 6:**
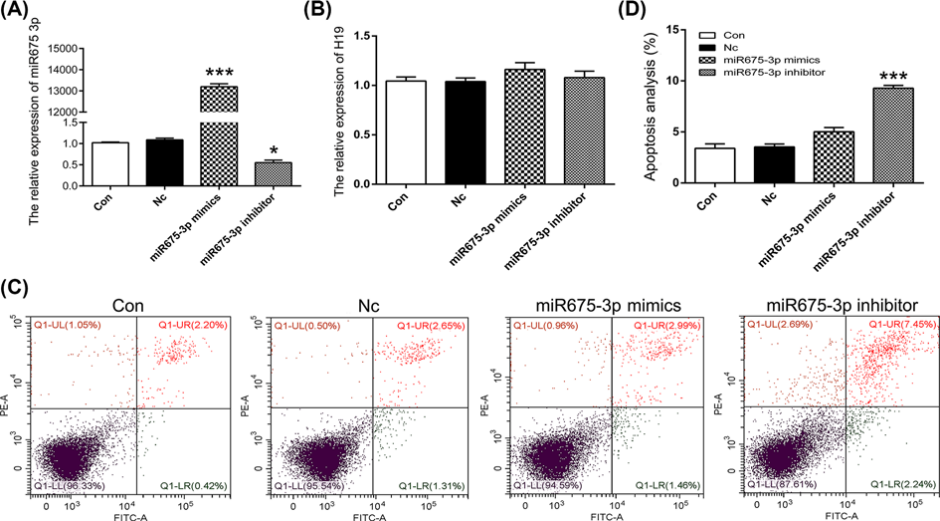
The expression pattern of miR675-3p in apoptosis The expression of miR675-3p (**A**) and *H19* (**B**) were analyzed by qPCR. (**C**) Apoptosis was analyzed after mutation of the m^6^A modification site. (**D**) Statistical analysis of apoptotic cell percentage. The data are presented as the mean ± SD. * (*P*<0.05) and *** (*P*<0.005) indicate statistically significant differences.

To further confirm that point mutations of m^6^A modification sites 129 bp upstream of miR675 induce cell apoptosis, we used L02 and MHCC97L cells. Results of Sanger sequencing showed identical point mutation patterns in both HEK293T and L02 cells (Figure 4A). qPCR results showed declined *H19* expression in the m^6^A-Mut group (Figure 4B). To investigate the effects of m^6^A modification on the expression patterns of miRNA, the expression of miR675 was analyzed. The results showed decreased expression of both miR675-3p and miR675-5p (Figure 4C). In addition, an increased cell apoptosis rate was observed in the m^6^A-Mut group (Figure 4D,E). To confirm that point mutations do induce cell apoptosis, the MHCC97L cell line (liver cancer cells) was used. As observed with HEK293T and L02 cells, a T–A base transversion was observed in MHCC97L cells (Figure 5A). Moreover, decreased expression of *H19*, miR675-3p, and miR675-5p was noted in MHCC97L cells in the m^6^A-Mut group (Figure 5B,C). An increased cell apoptosis rate was observed in MHCC97L cells after the introduction of point mutations as observed in HEK293T and L02 cells (Figure 5D,E). To further analyze miR675 expression patterns in apoptotic cells, HEK293T cells were treated with a mimic or inhibitor of miR675-3p. The results showed that reduced miR675-3p expression induced cell apoptosis (Figure 6). These results suggest that targeted point mutations of m^6^A modification sites 129 bp upstream of miR675 induced cell apoptosis through reduced expression of *H19*.

### m^6^A-related genes expression analysis by targeted point mutation

**Figure 7:**
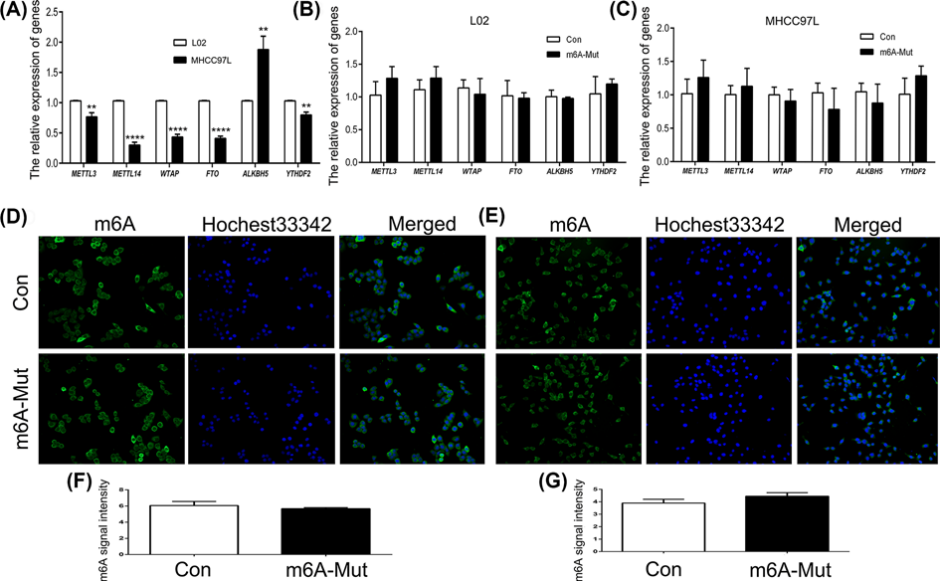
Expression pattern of m^6^A-related genes The expression of m^6^A-related genes was analyzed in L02 and MHCC97L cells (**A**). The expression of m^6^A-related genes analyzed by point mutations in L02 (**B**) and MHCC97L (**C**) cells. IF localization of m^6^A in L02 (**D**) and MHCC97L (**E**) cells. The fluorescence intensities of m^6^A were measured in L02 (**F**) and MHCC97L (**G**) cells. ** (*P*<0.01) and **** (*P*<0.001) indicate statistically significant differences.

We further explored the expression patterns of m^6^A-related genes after introducing point mutations in L02 and MHCC97L cells. qPCR results showed increased expression of *ALKBH5* and decreased expression of *METTL3, METTL14, WTAP, FTO*, and *YTHDF2* in MHCC97L cells compared with L02 cells (Figure 7A). This result suggested that compared with L02, there is abnormal expression of m6A genes in MHCC97L cells. However, the expression of m^6^A-related genes was not changed by point mutations in L02 and MHCC97L cells (Figure 7B,C). m^6^A expression level was analyzed using immunoflorescence (IF). The results showed that m^6^A expression was not altered in either L02 and MHCC97L cells (Figure 7D,E). Also, the statistical analysis confirmed the IF data (Figure 7F,G). These results indicated that targeted point mutations of miR675 did not change the global m^6^A expression levels in L02 and MHCC97L cells.

## Discussion

Previous reports have indicated that targeted point mutations result in C-to-T (BE3) or A-to-G (ABE7.10) conversions [18,20]. In this study, our results showed that a T–A base transversion occurred upstream of miR675 in HEK293T, L02, and MHCC97L cells. While the expected result was an A•T to G•C conversion, our data showed that an A•T to A•A conversion had occurred. These results indicate the partial effectiveness of the ABE7.10 system, which might have induced cell apoptosis. To confirm these data, we transfected the ABE7.10 system into A549 (lung cancer cells), SGC7901 (gastric cancer cells), and MHCC97H (liver cancer cells) cells. The result was in accordance with our previous data. In addition, a G-to-A conversion was observed which might have indicated incomplete mutation downstream of miR675. These results suggested the presence of the T–A base conversion pattern, which might have a role in cell apoptosis.

To further investigate the role of m^6^A modification in apoptosis, the expression patterns of *H19* and miR675 were analyzed. A previous study suggested that m^6^A modification was important for the expression of lncRNA and miRNA [21]. In our study, regions upstream and downstream of the m^6^A modification site of miR675 in the *H19* locus were evaluated. The results demonstrated that mutations in the regions upstream of the m^6^A modification site could suppress the expression of *H19* and miR675, whereas mutations in the regions downstream of the m^6^A modification site have no effect on the expression of *H19* and miR675. To confirm the expression patterns of *H19* and miR675 in apoptotic cells, overexpression and knockdown of *H19* and miR675 were examined. Previous reports showed that the expression of *H19* and miR675 was associated with cell apoptosis in cancer cells [22,23]. Our data suggest that reduced *H19* expression induced cell apoptosis through miR675, which was confirmed in human colorectal cancer cells [24]. These results indicate that regions upstream of the m^6^A modification site play a role in regulating the expression of *H19* and miR675 which can induce cell apoptosis.

There is evidence that reduced *H19* and miR675 expression led to increased p53 protein expression, which regulates cell apoptosis [25]. Our data showed that mutation of the regions upstream of the m^6^A modification site inhibited miR675 and *H19* expression, inducing cell apoptosis, possibly through the p53 protein. Moreover, an abnormal expression pattern of m^6^A-related genes was observed in liver cancer cells, which was in accordance with previous data [26]. A previous report suggested that *ALKBH5* overexpression promotes invasion and metastasis in gastric cancer cells [27]. Indeed, metastasis is the major factor for HCC. This indicates that *ALKBH5* expression may regulate the demethylated process and play a role in HCC metastasis. In addition, the global m^6^A expression was maintained after mutations of the regions upstream of the m^6^A modification site in L02 and MHCC97L cells. These results suggested that miR675 was regulated by m^6^A modification and has a role in *H19* expression, which in turn influences the fate of the cell.

## Conclusion

In summary, the ABE7.10 system resulted in efficient T–A base conversion of the m^6^A site upstream of miR675 in the *H19* locus. The expression of *H19* and miR675 was reduced by targeted point mutations. These mutations also induced cell apoptosis. Overall, our data suggest that m^6^A modification plays a role in gene expression and cell apoptosis.

## Supplementary Material

Supplementary Figure S1 and Tables S1-S4

## Abbreviations

ABE: adenine base editor
IF: immunofluorescence
lncRNA: long noncoding RNA
m^6^A: methylation of the adenine base at the nitrogen 6 position
PI: Propidium Iodide
qPCR: Quantitative Real-time PCR
