# Supplementary material for: Targeted point mutations of the m6A modification in miR675 using RNA-guided base editing induce cell apoptosis
Source: Biosci Rep. 2020 May 5;40(5):BSR20192933. doi: 10.1042/BSR20192933 (PMC7201566; doi:10.1042/BSR20192933)
Supplement: Supplementary Figure S1 and Tables S1-S4 [file BSR-2019-2933_supp.pdf]

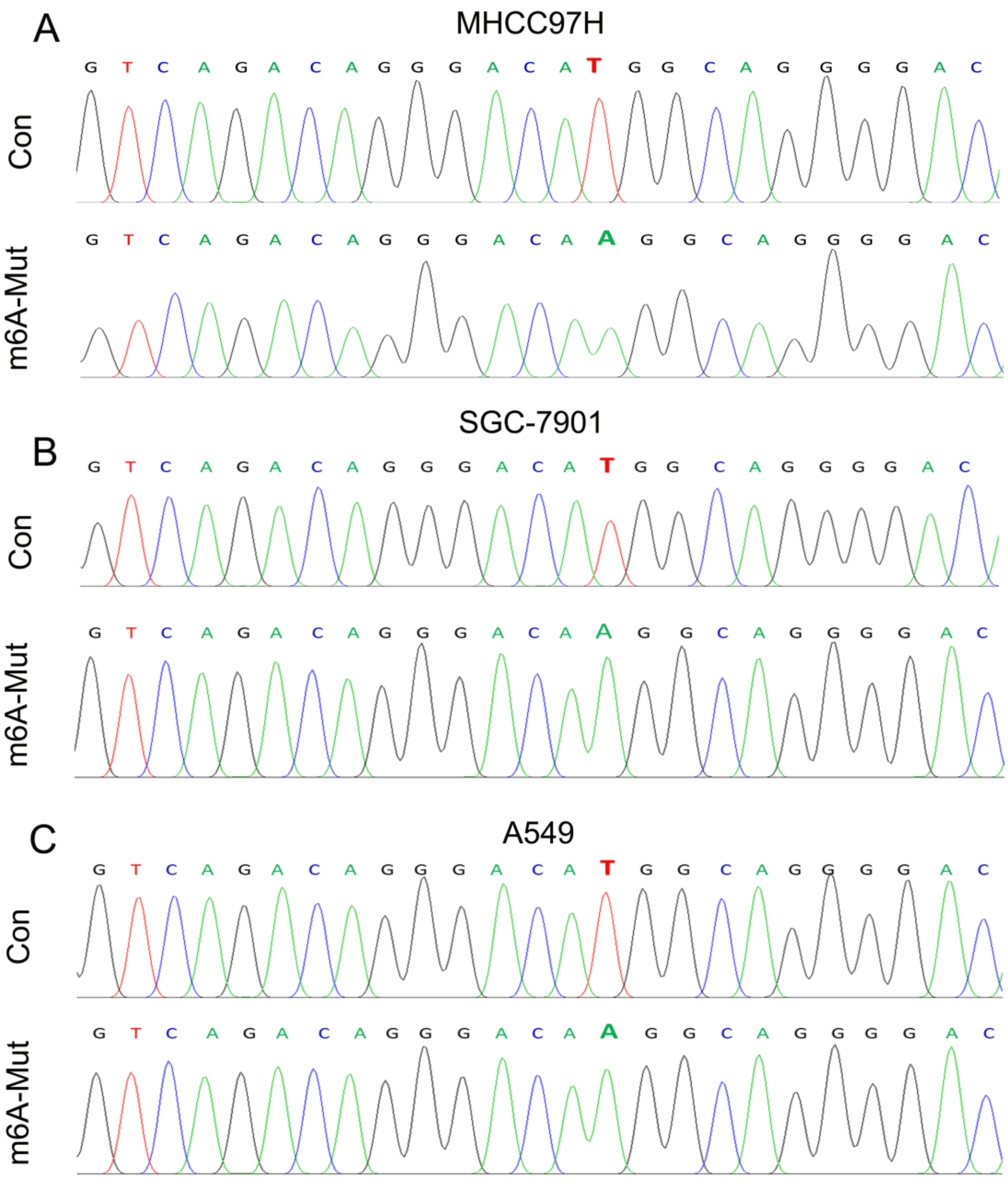
A
MHCC97H
G T C A G A C A G G G A C A T G G C A G G G G A C
Con
G T C A G A C A G G G A C A A G G C A G G G G A C
m6A-Mut
B
SGC-7901
G T C A G A C A G G G A C A T G G C A G G G G A C
Con
G T C A G A C A G G G A C A A G G C A G G G G A C
m6A-Mut
C
A549
G T C A G A C A G G G A C A T G G C A G G G G A C
Con
G T C A G A C A G G G A C A A G G C A G G G G A C
m6A-Mut

Figure S1. Sequencing analysis of the m^6A modification site. Mutated m^6A modification site in MHCC97H (A), SGC-7901 (B), and A549 (C) cells.

Table S1 Sequence of sgRNA

SgRNAs	Sequences (5'→3')	PAM
SgRNA1	F: AGGGACATGGCAGGGGACAC<br>R: GTGTCCCCTGCCATGTCCCT	AGG
SgRNA2	F: GGACACAAAACCCTCTAGCT<br>R: AGCTAGAGGGTTTTGTGTCC	TGG

Table S2 Sequence of miRNA

Genes	Annealing (°C)	Sequences (5'→3')
MIR675-5P	60	CTCAACTGGTGTCGTGGAGTCGGCAATTCAGTTGAGCACTGTGG
MIR675-3P	60	CTCAACTGGTGTCGTGGAGTCGGCAATTCAGTTGAGTGAGCGGT
U6	60	CGCTTCACGAATTTGCGTGTCAT

Table S3 Primers of miRNA for qRT-PCR analysis

Genes	Annealing (°C)	Primer sequences (5’→3’)
MIR675-5P	60	F: ACACTCCAGCTGGGTGGTGCGGAGAGGGCCC<br>R: CAGTGCGTGTCGTGGAGT
MIR675-3P	60	F: ACACTCCAGCTGGGCTGTATGCCCTCAC<br>R: CAGTGCGTGTCGTGGAGT
U6	60	F: GCTTCGGCAGCACATATACTAAAAT<br>R: CGCTTCACGAATTTGCGTGTCAT

Table S4 Primers of m6A related genes for qRT-PCR analysis

Genes	Annealing	Primer sequences (5’→3’)	Reference
METTL3	60	F: GAGGAGTGCATGAAAGCCAG<br>R: GGCCTCAGAATCCATGCAAG	(Feng et al. 2018)
METTL14	60	F: GACGGGGACTTCATTCATGC<br>R: CCAGCCTGGTCGAATTGTAC	(Feng et al. 2018)
ALKBH5	60	F: ACCCCATCCACATCTTCGAG<br>R: CTTGATGTCCTGAGGCCGTA	(Feng et al. 2018)
FTO	60	F: AGACACCTGGTTTGGCGATA<br>R: CCAAGGTTCCTGTTGAGCAC	(Feng et al. 2018)
YTHDF2	60	F: GAACCTTACTTGAGTCCACAG<br>R: GTAGGGCATGGCTGTGTCAC	(Melissa et al.2018)
WTAP	60	F: TGCGACTAGCAACCAAGGAA<br>R: ATCTCAGTTGGGCAACGCTC	(Melissa.et al.2018)
H19	60	F: AAAGACACCATCGG AACAGC<br>R: AGAGTCGTGGAGGCTTTGAA	(Hao et al. 2017)
GAPDH	60	F: TCTCCTCTGACTTCAACAGCGACA<br>R: CCCTGTTGCTGTAGCCAAATTCGT	(Feng et al. 2018)
